# Eagle syndrome in the pediatric population: A case report

**DOI:** 10.1002/ccr3.6148

**Published:** 2022-09-02

**Authors:** Zachary G. Tanenbaum, Stephanie Y. Johng, Keon M. Parsa, Mark E. Russo, Earl H. Harley

**Affiliations:** ^1^ Georgetown University School of Medicine Washington USA; ^2^ Department of Otolaryngology – Head and Neck Surgery Medstar Georgetown University Hospital Washington USA

**Keywords:** eagle syndrome, myofascial pain, otolaryngology, pediatric, styloid process elongation

## Abstract

Objectives: To present a rare case of Eagle Syndrome in a pediatric patient, reminding the medical community to keep this diagnosis on their differential.

## Graphical abstract

This case report demonstrates the need for otolaryngologists to maintain Eagle syndrome in their differential even when the presentation arises in pediatric patients.

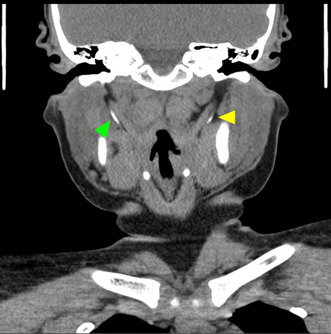

## 1 INTRODUCTION

Eagle syndrome (ES) is characterized by a triad of symptoms—dysphagia, cervical pain, and pharyngeal foreign body sensation. It is hypothesized that ES is caused by anatomical craniofacial variations impacting neurovascular structures adjacent to the glossopharyngeal nerve. Other associations include abnormal styloid process elongation or ossification of the stylohyoid ligament.^1^, ^2^ ES usually presents in women over the age of thirty and is associated with a history of tonsillectomy, adenoidectomy, and/or pharyngeal trauma. Due to the low prevalence of ES in the pediatric population, clinicians have a decreased propensity to incorporate ES onto their differential when a pediatric patient presents with debilitating orofacial pain and a presentation congruous to ES.

## 2 CASE REPORT

**FIGURE 1:**
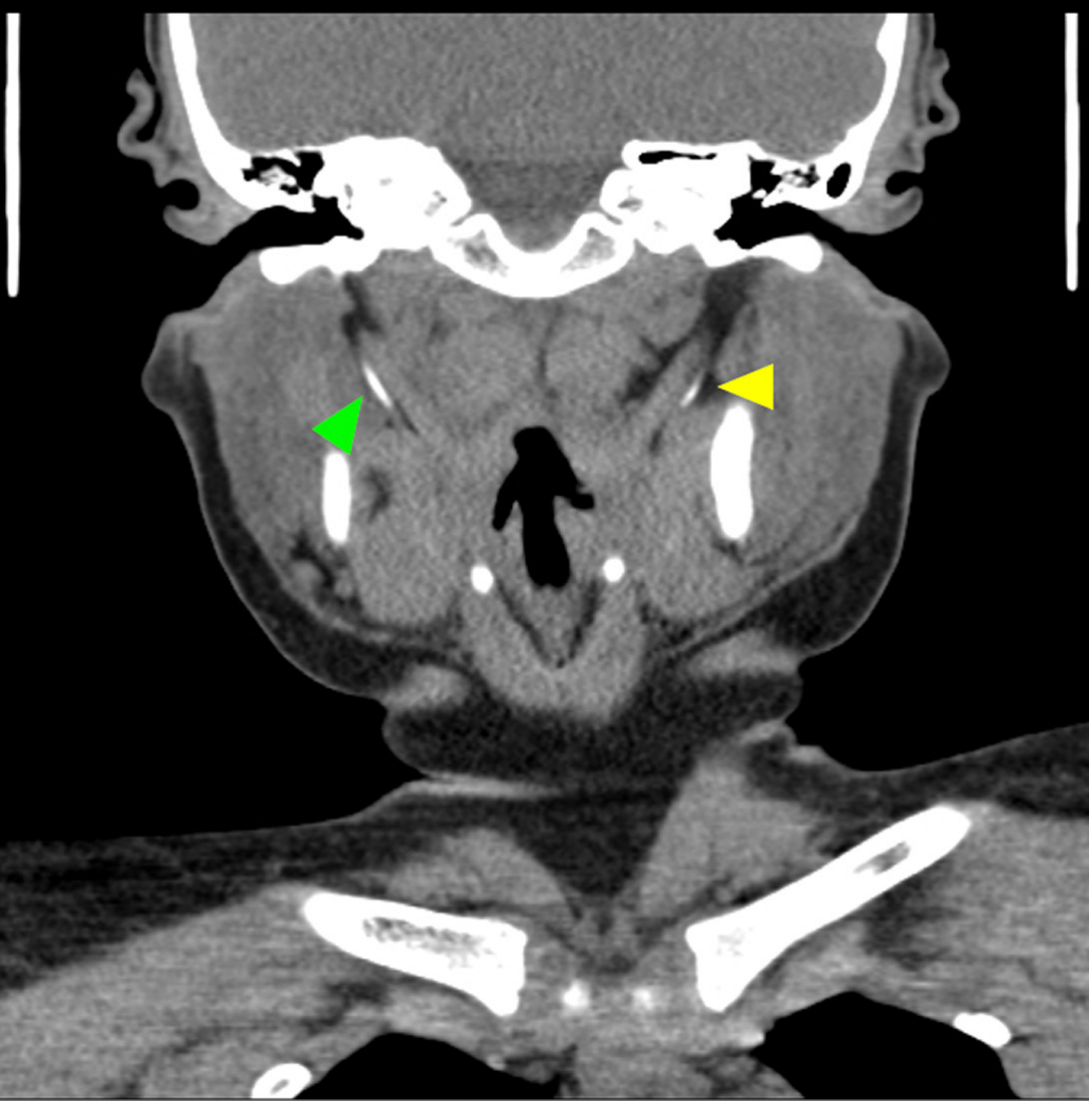
Coronal computed tomography (CT) without contrast revealing bilateral elongated styloid processes

A 10‐year‐old girl presented with complaints of intermittent throat pain and globus sensation. Her past surgical history was significant for adenotonsillectomy 3 years prior for obstructive sleep apnea (OSA). Since her surgery, the patient has complained of recurrent throat pain, predominantly on the left, that was consistently streptococcal negative. Although the patient denied coughing or congestion, her exam was notable for tenderness with palpation of the left tonsillar fossa area. A computed tomography (CT) revealed bilateral elongated styloid processes measuring 24 mm on the right and 25 mm on the left (Figure 1).

The patient then underwent endoscopy‐assisted transoral excision of the left styloid process. During the operation, the patient was placed under general anesthesia and prepped and draped in the usual fashion for tonsillectomy. A Crowe–Davis oral retractor was placed into the patient's oral cavity, and the patient was then suspended from the Mayo stand. The soft and hard palates were then palpated. Next, 2.5 cc of 0.25% Marcaine with epinephrine was injected into the left soft palate. A mucosal incision was made superior to the left anterior tonsillar pillar using the Colorado tip Bovie electrocautery. The zero‐degree Hopkins telescope was used to enhance visualization. Dissection was carried through the pharyngeal constrictors, and prestyloid fat was encountered. The distal tip of the styloid process was palpated and noted to be quite deep and posterolateral. A Kerasen was then used to remove a small piece of the distal tip of the styloid process. Mild venous bleeding was noted, and hemostasis was achieved using direct pressure and Surgiflo. 1 ml of 40 mg/mL of Kenalog was then injected near the styloid process. The incision was then closed with 3–0 Chromic in a running locking fashion. Next, 2 cc of additional 0.25% Marcaine with epinephrine was then injected around the incision line. The oral cavity was then irrigated, orogastric tube passed, and all contents of the patient's stomach were evacuated with suction. No surgical complications were noted throughout the surgery.

During follow‐up appointments, the patient reported left‐sided jaw pain at her one‐month, two‐month, and three‐month post‐operative visits. This was treated with non‐steroidal anti‐inflammatory drugs, heat, and a trial of Prednisone. The patient reported alleviation of symptoms shortly after her three‐month postoperative visit.

## 3 DISCUSSION

Pain radiating from the throat to the ear or mastoid process is a non‐specific complaint that can be attributed to a number of different conditions that should be excluded from the differential before considering ES—including temporomandibular joint dysfunction, glossopharyngeal neuralgia, and upper aerodigestive tract malignancies.^3^ Diagnosis is guided by history of present illness consistent with characteristic symptomatology, physical exam, and imaging. During the physical exam, a bony projection can often be felt via transpharyngeal palpitation of the tonsillar fossa. CT imaging is diagnostically important as it aids in styloid process visualization and allows for precise measurement of styloid process length, direction, anatomic variance, and ligament ossification.^3^

When first described in the literature by its namesake in 1937, adults with styloid processes longer than 25 mm were considered abnormal. Using this criterion, nearly 4% of adults would have an elongated styloid process, yet only 4% of those with elongation are symptomatic.^3^, ^4^ As calcification of the stylohyoid ligaments and elongation of the styloid process is age‐dependent and incredibly rare before adolescence, very little literature exists pertaining to the pediatric population.^4^ A thorough review of the literature demonstrates only five cases of pediatric ES to date. Although rare, it is imperative for otolaryngologists to maintain ES in their differential even when the presentation arises in pediatric patients.

While the etiology of ES has not yet been fully elucidated, clinical presentation can be attributed to symptomatic stretching of cranial nerves V, VII, IX, or X due to elongation of the styloid process or calcification of the stylohyoid ligament. Historically, case reports in the literature have cited past surgical trauma, including a history of tonsillectomy, as the initiating event causing calcification of the ligament. Interestingly, recent literature has also reported occasional cases of ES in adult patients who have never been operated on for tonsillectomy and who have neither styloid process elongation nor ossification.^1^, ^2^, ^3^, ^5^

Treatment options can be both surgical and medical. Pharmacologic treatments include NSAIDs, antidepressants, and/or transpharyngeal infiltration of corticosteroids or anesthetics into the tonsillar fossa. Surgical treatments can be intraoral or extraoral and often entail a partial styloidectomy.^1^ The intraoral approach has the advantages of shorter operative times and better aesthetic outcomes due to the lack of scar formation. However, there is greater risk of intraoperative nerve injury due to limited exposure, which can be abated by an endoscopic assisted approach.^1^

## 4 CONCLUSION

As demonstrated by this unique case report, it is possible for Eagle syndrome to arise in the pediatric population. Therefore, it is imperative that otolaryngologists maintain ES in their differential even when the presentation arises in pediatric patients.

## AUTHOR CONTRIBUTIONS

Zachary Tanenbaum, MD: Responsible for writing the manuscript, editing, and submission. Stephanie Johng, MS: Responsible for writing the manuscript. Keon Parsa, MD: Responsible for manuscript editing. Mark Russo, MD: Responsible for manuscript oversite. Earl H Harley, MD: Responsible for oversite and mentorship of the report.

## CONFLICT OF INTEREST

None.

## CONSENT

Written informed consent was obtained from a parent/guardian to publish this report in accordance with the journal's patient consent policy.

## Data Availability

The data that support the findings of this study are available on request from the corresponding author. The data are not publicly available due to privacy or ethical restrictions.
